# Non-severe hypoglycemia in type 1 diabetes: a randomized crossover trial comparing two quantities of oral carbohydrates at different insulin-induced hypoglycemia ranges

**DOI:** 10.3389/fendo.2023.1186680

**Published:** 2023-06-02

**Authors:** Nadine Taleb, Véronique Gingras, Ran Cheng, Valérie Parent, Virginie Messier, Danijela Bovan, Azadeh Shohoudi, Anne-Sophie Brazeau, Rémi Rabasa-Lhoret

**Affiliations:** ^1^ Montreal Clinical Research Institute, Montreal, Canada; ^2^ Biomedical Sciences Department, Faculty of Medicine, Université de Montréal, Montreal, Canada; ^3^ Endocrinology Division, Centre hospitalier de l’Université de Montréal, Montreal, Canada; ^4^ Research Centre, CHU Sainte-Justine, Montreal, Canada; ^5^ Department of Nutrition, Faculty of Medicine, Université de Montréal, Montreal, Canada; ^6^ School of Human Nutrition, McGill University, Montreal, Canada; ^7^ Montreal Diabetes Research Center, Montreal, Canada

**Keywords:** non-severe hypoglycemia, hypoglycemia treatment guidelines, type 1 diabetes, oral carbohydrate, subcutaneous insulin injection

## Abstract

**Aims:**

Non-severe hypoglycemia (NS-H) is challenging for people living with type 1 diabetes (PWT1D) and often results from relative iatrogenic hyper-insulinemia. Current guidelines recommend a one-size-fits-all approach of 15–20 g of simple carbohydrates (CHO) every 15 min regardless of the triggering conditions of the NS-H event. We aimed to test different amounts of CHO to treat insulin-induced NS-H at various glucose ranges.

**Methods:**

This is a randomized, four-way, crossover study involving PWT1D, testing NS-H treatment outcomes with 16 g vs. 32 g CHO at two plasma glucose (PG) ranges: A: 3.0–3.5 mmol/L and B: <3.0 mmol/L. Across all study arms, participants consumed an additional 16 g of CHO if PG was still <3.0 mmol/L at 15 min and <4.0 mmol/L at 45 min post-initial treatment. Subcutaneous insulin was used in a fasting state to induce NS-H. Participants had frequent venous sampling of PG, insulin, and glucagon levels.

**Results:**

Participants (*n* = 32; 56% female participants) had a mean (SD) age of 46.1 (17.1) years, had HbA1c at 54.0 (6.8 mmol/mol) [7.1% (0.9%)], and had a diabetes duration of 27.5 (17.0) years; 56% were insulin pump users. We compared NS-H correction parameters between 16 g and 32 g of CHO for range A, 3.0–3.5 mmol/L (*n* = 32), and range B, <3.0 mmol/L (*n* = 29). Change in PG at 15 min for A: 0.1 (0.8) mmol/L vs. 0.6 (0.9) mmol/L, *p* = 0.02; and for B: 0.8 (0.9) mmol/L vs. 0.8 (1.0) mmol/L, *p* = 1.0. Percentage of participants with corrected episodes at 15 min: (A) 19% vs. 47%, *p* = 0.09; (B) 21% vs. 24%, *p* = 1.0. A second treatment was necessary in (A) 50% vs. 15% of participants, *p* = 0.001; (B) 45% vs. 34% of participants, *p* = 0.37. No statistically significant differences in insulin and glucagon parameters were observed.

**Conclusions:**

NS-H, in the context of hyper-insulinemia, is difficult to treat in PWT1D. Initial consumption of 32 g of CHO revealed some advantages at the 3.0–3.5 mmol/L range. This was not reproduced at lower PG ranges since participants needed additional CHO regardless of the amount of initial consumption.

**Clinical trial registration:**

ClinicalTrials.gov, identifier NCT03489967.

## Introduction

People living with type 1 diabetes (PWT1D) are faced with increased risks of hypoglycemia and its negative impacts when trying to achieve optimal glucose management (1, 2). Diabetes societies classify hypoglycemia as severe when another person must assist to treat it or non-severe when the patient can self-manage it. Recent studies using glucose sensors reported 1.5 h/day spent in hypoglycemia and 0.76 daily treated non-severe hypoglycemic (NS-H) episodes (3, 4). It is essential to optimize NS-H treatment to bring blood glucose to safe levels in a fast and efficient manner without causing rebound hyperglycemia. NS-H treatment guidelines stem from old clinical studies and expert opinions and have remained unchanged for decades. They recommend a one-size-fits-all approach of consuming 15–20 g of simple CHO every 15 min until correction (15 g/15 min rule) (1, 5–7). Same recommendations apply regardless of many factors potentially influencing the response to treatment such as glucose levels during treatment, estimated level of hyper-insulinemia, modality of insulin therapy, body weight, and activity level (8).

Recent studies and review reports have questioned the 15 g/15 min rule and have required extensive research studies to investigate the most efficient treatment strategies according to different NS-H settings and conditions (8). The aim would be to fine-tune NS-H treatment guidelines for PWT1D (4, 8–10). An observational study, in free-living conditions, of PWT1D’s practices showed a mean initial intake of 32 ± 24 g CHO to correct NS-H episodes (4). According to the current guidelines, this translates into 73% of participants overtreating their NS-H episodes (more than 20 g within the first 15 min) (4). However, this practice could equally reflect the inefficiency of the current guidelines to meet PWT1D’s needs in treating their NS-H (8, 9).

Thus, we hypothesized that a higher initial CHO consumption than the recommended 15–20 g might be more effective in correcting NS-H and reduce the need to repeated treatments. We aimed to test this approach at two hypoglycemia ranges.

## Methodology

### Study design

This is an open-label, randomized, four-way, crossover study investigating 16 g vs. 32 g of CHO to correct insulin-induced NS-H at two hypoglycemia ranges, 3.0–3.5 mmol/L and <3.0 mmol/L, in PWT1D.

### Study subjects

Eligible subjects were adults (≥18 years of old) with a clinical diagnosis of T1D for at least 1 year, treated with either multiple daily insulin injections (MDI) or continuous subcutaneous insulin injection (CSII) and a glycated hemoglobin HbA1c ≤ 89 mmol/mol (≤10%). Exclusion criteria included clinically significant microvascular complications, recent (<3 months) acute macrovascular event, significant cardiac rhythm abnormality, abnormal blood panel and/or anemia (hemoglobin < 100 g/L), ongoing pregnancy or breastfeeding, severe hypoglycemic episodes within 1 month of screening, or additional health problems that could affect the participation to the study as per medical assessment of the recruiting physician.

The ethics committee at Montreal Clinical Research Institute approved the study that was conducted according to the Declaration of Helsinki. All participants provided written informed consent. Subjects were recruited at the Montreal Clinical Research Institute (IRCM) through the T1D clinic and from a pool of participants who had participated in previous study protocols. The study followed a block balanced randomization and envelopes were not opened until a participant had met the inclusion criteria and signed the informed consent.

### Study procedures

Participants installed a glucose sensor (Dexcom G4 or G5 Platinum, Dexcom, USA) 24–48 h before study visits and installation training was offered for those unfamiliar with the technology. At least 6-day intervals separated study visits. The day before each study visit, participants had to refrain from exercise and alcohol consumption and reduced their nighttime basal insulin by 10% to minimize nocturnal hypoglycemia risks. Upon arrival to the testing centre, Dexcom data were uploaded and verified. A test was reported in the case of nocturnal hypoglycemia (two separate periods of 20 min or one single period exceeding 40 min with glucose levels < 3.5 mmol/L). At 7:30 a.m., participants had subcutaneous rapid insulin analog injection according to body weight and starting glucose level to induce hypoglycemia. Insulin doses were 0.13 U/kg for plasma glucose (PG) 10.0–15.0 mmol/L, 0.10 U/kg for PG 7.0–9.9 mmol/L, and 0.08 U/kg for PG 4.0–6.9 mmol/L. Intravenous catheters were installed to collect venous blood samples every 15 min for PG above 5 mmol/L, every 10 min for PG above 4 mmol/L, and then every 5 min until hypoglycemia threshold. Then, ® glucose tablets for 16 g or 32 g were consumed and PG-T0 was defined as time 0 post-initial CHO consumption. Blood samples were later collected at 5, 10, 15, 20, 30, 45, and 60 min. Participants consumed a second treatment with 16 g of CHO at 15 min if PG-T15 was < 3.0 mmol/L and at 45 min if PG-T45 was < 4.0 mmol/L.

All blood samples were centrifuged immediately, and PG was measured using YSI 2300 STAT Plus analyzer (Yellow Springs, OH, USA). Plasma samples were stored at −80°C until subsequent measurement of insulin and glucagon using respective immunological assays (Millipore, Billerica, MA, USA).

### Statistical analysis

We conducted analyses separately for each hypoglycemia range to compare the effect of initial treatment of NS-H with 16 g vs. 32 g CHO. The primary outcome was the change in PG at 15 min (Delta-PG-15) after CHO consumption. Secondary outcomes are listed in the *Results* section. We assumed that a Delta-PG-15 after hypoglycemia treatment with 16 g of CHO would be 0.82 ± 1.4 mmol/L and a difference between 16 g of CHO vs. 32 g of CHO would be 1.1 mmol/L higher with 32 g (11, 12). Under these assumptions, using a corrected type I error of 0.025 (0.05/2), the sample size was 29 participants for a power of 80%. To account for potential 10% dropout rates, we calculated that 32 participants should be recruited in total. Calculations were done using SAS 9.4 power procedure for paired design. We applied a linear mixed effect model (LMEM) with Gaussian family and identified link function, with the intervention, treatment sequence, period, and PG-T0 entered as fixed effect covariates and subject nested within sequence as a random effect covariate. The model was suited for repeated observations, i.e., adjustments for patient-level intra-correlation. Since time variables have positive values that are often not normally distributed, we used non-parametric bootstrap procedures to estimate parameters and their 95% confidence interval when applicable.

Finally, all hypoglycemic event data (*n* = 122) were pooled for secondary analysis of Delta-PG-15-min. Linear regression models were built with the following variables: initial CHO consumption, sex, diabetes duration, body mass index, PG-T0, rate of change of PG, rate of change of insulin, and rate of change of glucagon with adjustment for within-subject correlation. The final model was selected based on the best fit considering Akaike information criteria, Bayesian information criteria, R-squared, mean standard error, intra-class correlation coefficient, and dispersion parameter sigma calculations. We used R software version 3.1.2 and SAS 9.4 for all data analysis. A *p*-value < 0.05 was considered statistically significant.

## Results

**Figure 1:**
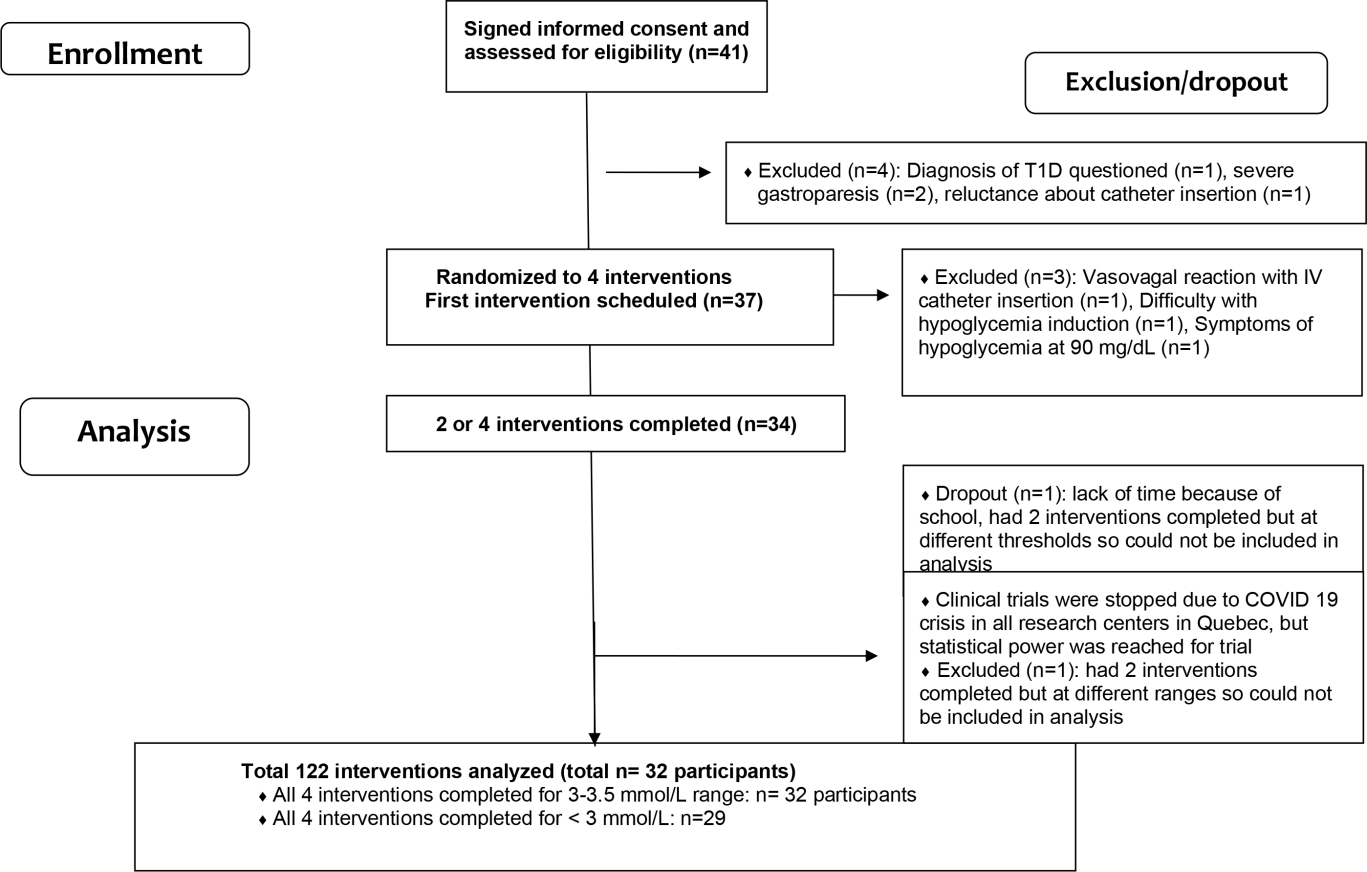
Trial flowchart.

Participants included in the analysis (*n* = 32, 56% female participants and 56% insulin pump users) were on average (SD) 46.1 (17.1) years old with an HbA1c of 54 (6.8) mmol/mol [7.1 (0.9)%], a diabetes duration of 27.5 (13) years, a body mass index of 25.7 (3.4) kg/m^2^, and a daily insulin dose of 0.5 (0.2) U/kg/day (**Table 1**). All 32 participants completed the two arm trials of 16 g vs. 32 g of CHO at the range of 3–3.5 mmol/L; 29 of the participants also completed them at PG < 3 mmol/L. Recruitment details are in a study flowchart (**Figure 1**).

**Table 1:** Baseline characteristics of the participants.

*n* = 32 (18 women and 14 men)		
	Mean (SD)	Minimum/Maximum
Age, years	46.1 (17.1)	19.5/71.5
HbA1c, mmol/mol %	54 (6.8) 7.1 (0.9)	41.8/75.3 5.5/9.9
Diabetes duration, years	27.5 (17.0)	3/63
BMI, kg/m^2^	25.7 (3.4)	20.6/32.7
Total insulin dose, U/kg/day	0.5 (0.2)	0.2/0.9
Insulin therapy, *n*: CSII/MDI	18/14	N/A

**Figure 2:**
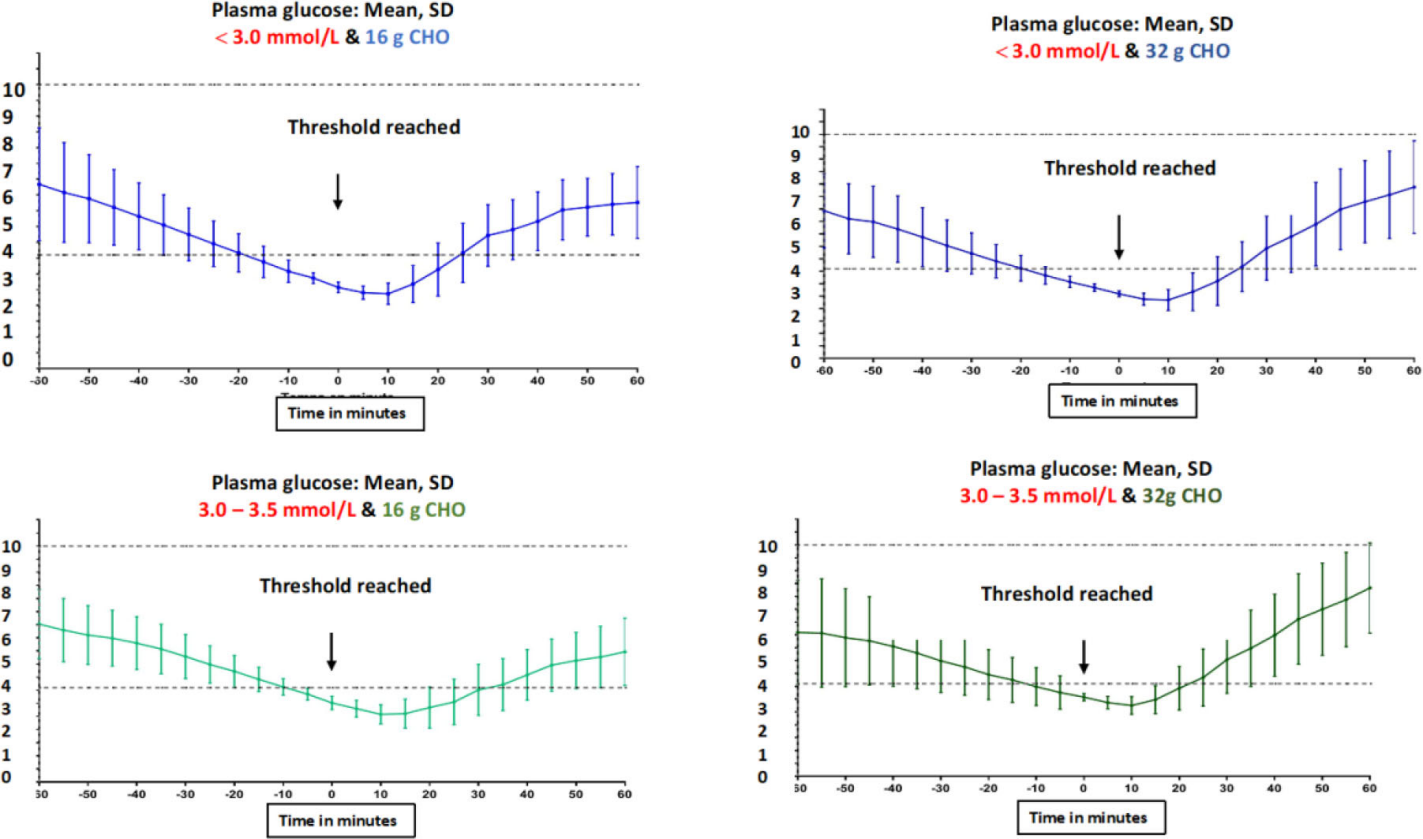
Plasma glucose profiles starting at 60 min before hypoglycemia treatment level was reached until 60 min after CHO consumption for the four trial arms.

Plasma glucose profiling for each of the four trial arms is shown in **Figure 2**. Mean PG at 60 min before hypoglycemia treatment shows inter-individual variability as reflected by wide SDs. The variability was brought to minimum upon reaching the target hypoglycemia ranges. Thereafter, the variability widened again in the next 60 min post-treatment, reflecting differences in inter-individual responses to CHO consumption.

We report the comparison of 16 g vs. 32 g of CHO for the PG range 3.0–3.5 mmol/L in 32 participants. Delta-PG-15-min reached 0.1 (0.8) mmol/L vs. 0.6 (0.9) mmol/L, *p* = 0.02. A second treatment was needed for 50% vs. 15% of subjects, *p* = 0.001. There were 0 vs. 4 events with PG >10 mmol/L at any time in the 60 min following correction (maximum reached PG level was 11.8 mmol/L) (**Table 2**).

**Table 2:** Outcomes of hypoglycemia treatment with 16 vs. 32 g oral CHO.

Outcomes of hypoglycemia treatment at PG 3.0–3.5 mmol/L (*N* = 32)			
Initial treatment	16 g CHO	32 g CHO	*p*-value
PG-T0, mmol/L	3.1 (0.4)	3.2 (0.3)	0.3
Insulin bolus, U/kg	0.12 (0.21)	0.12 (0.22)	0.6
Changes of PG from PG-T0, mmol/L at 15 min *	0.1 (0.8)	0.6 (0.9)	**0.02**
Participants with hypoglycemia corrected at 15 min (PG ≥ 4 mmol/L), %	19%	47%	0.09
2nd CHO treatment, *n* (%)	16 (50%)	5 (15%)	**0.001**
Time spent in hypoglycemia < 4 mmol/L, min	48.1 (13.4)	40.2 (14.0)	**0.01**
PG, rate of change, mmol/L/min	−0.05 (0.02)	−0.05 (0.03)	0.5
Plasma insulin at initial treatment, pmol/L	290 (165)	260 (207)	0.1
Plasma insulin, rate of change, pmol/L/min	0.21 (0.17)	0.18 (0.22)	0.3
Plasma glucagon at treatment, ng/L	42.6 (19.0)	39.3 (18.4)	0.4
Plasma glucagon, rate of change, ng/L/min	−0.13 (0.24)	0.02 (0.65)	0.2
Outcomes of hypoglycemia treatment at PG < 3 mmol/L (*N* = 29)			
Initial treatment	16 g CHO	32 g CHO	*p*-value
PG-T0, mmol/L	2.7 (0.2)	2.6 (0.3)	0.6
Insulin bolus, U/kg	0.14 (0.25)	0.14 (0.33)	0.8
Changes of PG from PG-T0, mmol/L at 15 min *	0.8 (0.9)	0.8 (1.0)	1.0
Participants with hypoglycemia corrected at 15 min (PG ≥ 4 mmol/L), %	21%	24%	1.0
2nd CHO treatment 16 g, *n* (%)	13 (45%)	10 (34%)	0.37
Time spent in hypoglycemia < 4 mmol/L, min	51.7 (12.4)	52.2 (15.1)	0.8
PG, rate of change, mmol/L/min	−0.06 (0.03)	−0.05 (0.02)	0.5
Plasma insulin at initial treatment pmol/L	275.7 (183.3)	278.4 (170.1)	0.8
Plasma insulin, rate of change, pmol/L/min	0.17 (0.18)	0.17 (0.25)	1.0
Plasma glucagon at treatment, ng/L	39.6 (16.9)	44.8 (17.7)	0.2
Plasma glucagon, rate of change, ng/L/min	−0.16 (0.50)	−0.03 (0.31)	0.2

Data presented as mean (SD) or n (%). *Primary endpoint. PG-T0 is plasma glucose at time 0, which is at the end of glucose tablets consumption. 2nd CHO treatment given at 15 min if PG still < 3 mmol/L and at 45 min if PG < 4 mmol/L. Rate of change of PG, insulin, and glucagon are calculated from hypoglycemia induction until first CHO consumption. Statistically significant p values were put in bold.

We compare the consumption of 16 g vs. 32 g CHO intake for PG range < 3 mmol/L in 29 participants. Delta-PG-15-min reached 0.8 (0.9) mmol/L vs. 0.8 (1.0) mmol/L, *p* = 1.0; 45% vs. 34% of subjects needed a second treatment, *p* = 0.37. There were 0 vs. 2 events with PG >10 mmol/L at any time in the 60 min following correction (maximum PG level was 11.0 mmol/L) (**Table 2**).

At both hypoglycemia ranges, insulin boluses per kg of body weight used to induce hypoglycemia and plasma insulin and glucagon levels upon reaching hypoglycemia thresholds were comparable between arms (**Table 1**). No severe hypoglycemia or neuro-glycopenic adverse events were recorded at any intervention.

According to the best-fit regression model built for pooled data, significant predictors (*β* estimate; 95% CI, *p*-value) of delta-PG-15-min included initial CHO consumption (0.02; 0.002 to 0.03; *p* = 0.03), PG-T0 (−0.70; −1.1 to −0.2; *p* < 0.01), and rate of PG decrease (9.7; 0.6 to 17.5; *p* = 0.03). A trend was noted for rates of insulin increase (−0.9; −1.7 to 0.06; *p* = 0.05). No statistically significant effects were observed for sex, diabetes duration, and BMI.

## Discussion

For insulin-induced NS-H, an initial CHO intake of 32 g compared to 16 g of CHO at the 3.0–3.5 mmol/L hypoglycemic range showed some benefits. This resonates with real-life practices of PWT1D who consumed averages of 32 g of CHO (4). There was less time in hypoglycemia (8 min less) and reduced need to repeat treatment (one-fifth vs. one-half of subjects). Below the 3 mmol/L threshold, a clear advantage was not maintained with higher initial CHO consumption. Although one-third vs. one-half of the participants needed a second treatment, this observation was not statistically significant. Overall, our data confirm the difficulty of treating insulin-induced NS-H for PWT1D.

In our study, an increase in PG at 15 min post-treatment with 16 g of CHO was similar to that reported by a recent study (0.85 mmol/L) (9). However, this is lower than reported in older studies referred to in current guidelines. PG levels rose by 1.1 to 1.8 mmol/L with similar oral CHO during intravenously induced NS-H experiments in older trials (6, 7). Differences in type and mode of insulin administration for hypoglycemia induction could explain these differences. Older studies induced hypoglycemia with intravenous regular human insulin in contrast to subcutaneous rapid insulin analogs in our trial. A subcutaneous route for insulin administration, like in our study, results in wider inter-patient insulin absorption variability and subsequently affects glycemia. Nevertheless, it is more representative of patients’ real-life experiences in comparison to intravenous insulin.

As previously mentioned, we chose 32 g of CHO for initial consumption based on averages reported in real-life patients’ practices (4). Studies testing different amounts of CHO are scarce. McTavish et al. tested 15 g or 0.2 g/kg or 0.3 g/kg body weight in the form of glucose tablets for capillary glucose < 4 mmol/L, to be repeated every 10 min until hypoglycemia correction (14). Consuming 0.3 g/kg CHO (median 24 g at 3.3 mmol/L) was most effective in resolving hypoglycemia and necessitated repeated consumption in 29% of episodes (14). In our study, 32 g of CHO at a PG range of 3.0–3.5 mmol/L seemed efficient, necessitating second treatment in only 15% of subjects (vs. 35% to 50% in other arms). Our criteria for repeated treatment were less strict than those of McTavish et al. (longer wait and lower cutoff). We have opted for a second treatment with 16 g at 15 min if PG was still inferior to 3 mmol/L, instead of 4 mmol/L, as a safety measure. As such, our design gave a chance to explore the effect of 32 g of CHO, knowing that the absorption rate of CHO could differ and be longer in certain individuals. This design minimized the risk of rebound hyperglycemia with excessive repetitive treatment CHO consumption. In both trials, higher initial CHO than the recommended 16–20 g did not result in clinically significant rebound hyperglycemia. It is noteworthy that sensor glucose values often lag behind plasma levels, especially in hypoglycemia ranges (15). Accordingly, we chose a plasma glucose threshold lower than 3.5 mmol/L rather than 4 mmol/L for our study.

This potential advantage of an initial 32 g of CHO at a PG range of 3.0-3.5 mmol/L was not seen at lower hypoglycemia at which more intense and unpleasant symptoms were reported (data not shown) (16). One could hypothesize that it takes longer to replete PG once lower hypoglycemia ranges had been reached, regardless of initial CHO amounts, possibly due to higher uptake of exogenous glucose by energy-depleted organs at lower thresholds. It would be interesting to explore disposal rates of exogenous oral CHO at different hypoglycemia thresholds in PWT1D, but this was beyond the objectives of our current trial.

The pooled data were limited by its secondary analysis nature but it leads to interesting observations. It demonstrated that both absolute glucose levels and its rates of change (data that can be obtained with glucose sensors) and possibly rates of change of plasma insulin levels (research in progress designing insulin sensors) were positively associated with change of PG at 15 min (11). If integrated in future artificial intelligence algorithms, these parameters could suggest adapted amounts and timings of CHO consumption (11, 16). A higher initial CHO consumption of 32 g was positively associated with delta-PG-15.

Mini-doses of glucagon could be an alternative to oral CHO in NS-H, especially to avoid additional caloric intake with the increased prevalence of excess weight in PWT1D (17). Yet, research is still emerging in this area (12). A small study in PWT1D showed that 75, 150, and 300 μg of injectable glucagon could raise glucose levels after an overnight fast (13). However, efficacy was less at glucose levels < 4 mmol/L post-subcutaneous insulin boluses. At 20 min after glucagon injections, glucose levels increased only by 0.2 to 0.4 mmol/L and subjects reported nausea at the 150-μg dose (13). Thus, the action of mini-doses of glucagon was blunted after an insulin bolus but preserved for hypoglycemia during low circulating insulin levels (13). Mini-doses of glucagon might be more practical with nasal forms in comparison with injectable ones but lower doses than 3 mg should be available (12). Importantly, with all forms of glucagon, the efficiency, stability, and safety of repeated administration for the treatment of NS-H would need to be extensively tested and proven (18).

Our study had some limitations. Subcutaneous insulin could result in inter-patient variability due to differences in absorption, but it is more representative of real-life conditions than the historical use of the intravenous route. These results need replication during spontaneous hypoglycemia episodes in diverse free-living conditions using glucose sensors or capillary values and in pediatric populations. Alternate ways to treat NS-H should be investigated. We strongly believe that CHO intake at glucose thresholds higher than 4 mmol/L to prevent reaching serious hypoglycemia levels needs to be investigated.

In conclusion, this study has confirmed treatment difficulties of insulin-induced NS-H with the current guidelines. While it did not aim to change those guidelines, it added one important piece to the puzzle. Advantages of higher initial CHO consumption in some circumstances and treatment at higher glucose thresholds or ranges should be further reproduced in other trials and settings. This study adds to the scarce literature needed to fine-tune the treatment approach for this daily struggle for PWT1D and asserts the need to conduct other trials to cover various clinical scenarios and conditions of NS-H (8).

## Data availability statement

The data analyzed in this study is subject to the following licenses/restrictions: Granular data can be made available upon direct request to the senior author. Requests to access these datasets should be directed to remi.rabasa-lhoret@ircm.qc.ca.

## Ethics statement

The studies involving human participants were reviewed and approved by the Ethics committee at Montreal Clinical Research Institute. The patients/participants provided their written informed consent to participate in this study.

## Author contributions

NT, VG, VM, and RR-L designed the study. NT, VP, and DB conducted data collection. NT and AS conducted data analysis. NT, A-SB, VG, RC, and RR-L helped in data interpretation. NT drafted the manuscript, which was critically revised by VP, VG, A-SB, AS, DB, RC, and RR-L. All authors revised the final draft and approved its contents. RR-L is the principal guarantor of this work.
